# Analysis of postmarketing safety data for proton-pump inhibitors reveals increased propensity for renal injury, electrolyte abnormalities, and nephrolithiasis

**DOI:** 10.1038/s41598-019-39335-7

**Published:** 2019-02-19

**Authors:** Tigran Makunts, Isaac V. Cohen, Linda Awdishu, Ruben Abagyan

**Affiliations:** Skaggs School of Pharmacy and Pharmaceutical Sciences, University of California, San Diego, La Jolla, CA 92093-0747 USA

## Abstract

Proton pump inhibitors, PPIs, are widely prescribed and sold globally. Although initially intended for time-limited treatment of acute disorders, such as gastric ulcers and esophagitis, PPIs are now commonly used for prolonged durations and are considered safe for over the counter access. Recent studies have raised concern over associations between PPI use and acute kidney injury, chronic kidney disease, end-stage renal disease, and electrolyte abnormalities. The growing concern over potentially serious adverse drug reactions warrants an evaluation of post marketing surveillance data. In this study of over ten million FDA Adverse Event Reporting System records, we provided evidence of kidney injury and electrolyte imbalances in an alarming number of patients taking PPIs. Additionally, we assessed differences between specific PPIs and observed significant electrolyte and renal abnormalities for each individual drug with varying magnitudes.

## Introduction

The World Health Organization includes proton pump inhibitors (PPIs) in the list of essential medicines and health products^1^. PPIs have demonstrated superior efficacy to histamine-2 receptor antagonists (H2RAs) in treatment of acid-related disorders and have replaced the H2RAs^2,3^. The current indications include treatment of gastroesophageal reflux disease, non-steroidal anti-inflammatory drug (NSAID) and *Helicobacter pylori* induced ulcers, duodenal ulcers, erosive esophagitis, and other pathological hypersecretory conditions, including Zollinger-Ellison syndrome^4,5^ (see Supplement-Appendix A for a more comprehensive indication list reported to FDA) and are now one of the most widely utilized medications^6^. The superior efficacy is credited to the mechanism of action. All currently marketed PPIs inhibit the hydrogen pump H +/K+ ATPase irreversibly, preventing the last and rate-limiting step in acid secretion by parietal cells in the stomach^7,8^. There are currently six Food and Drug Administration (FDA) approved PPIs: rabeprazole, lansoprazole, pantoprazole, esomeprazole, omeprazole, and dexlansoprazole. These were sequentially developed due to varying pharmacokinetic parameters, such as extended plasma half-life, routes of administration, and drug interactions^9,10^. The most common PPI adverse reactions (ADRs) are mild and include headache, nausea, stomach pain, diarrhea, vomiting, and flatulence. Serious allergic reactions include rash, facial swelling, throat tightness, and difficulty breathing^11^. Generally considered safe, PPIs are now commonly used for prophylaxis and sold over the counter in most of the industrialized countries, including the United States, with annual prescription and over the counter sales exceeding fourteen billion dollars anually^12^.

Recently, PPI use has come under scrutiny due to growing evidence of renal, cardiovascular, autoimmune and neurologic adverse effects. New data has revealed associations with myocardial infarction^13^, Clostridium difficile-associated diarrhea^14^, community acquired pneumonia^15^, bone fractures^16^, subacute cutaneous lupus erythematosus^17,18^, Alzheimer’s dementia^19,20^, and kidney injury^21–29^. Here we evaluated the frequencies of reported adverse events related to kidney injury and electrolyte disturbances in patients taking PPIs. We also compared the magnitude of the effects for individual PPIs.

## Results

### PPI “monotherapy”-related renal and electrolyte ADRs

**Figure 1:**
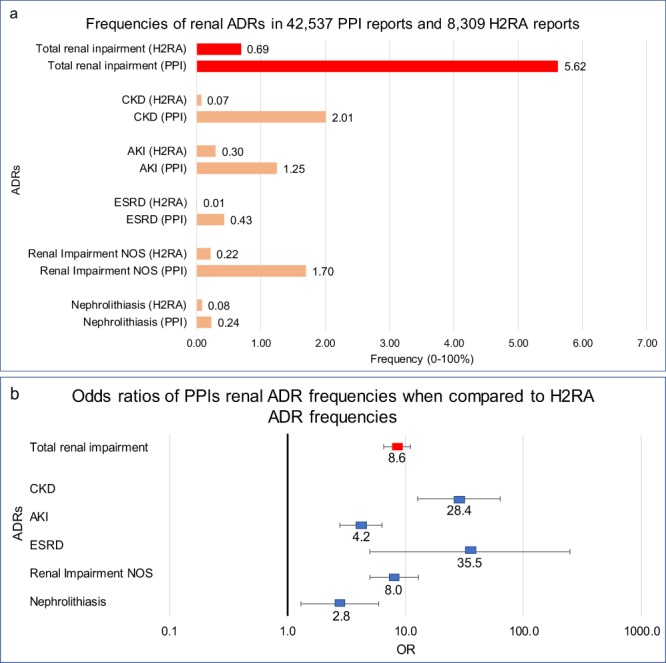
Frequencies and odds ratios (ORs) of renal adverse drug reactions (ADRs). (**a**) Frequencies of renal adverse events for patients in FAERS/AERS who took PPIs (n = 42,537) and H2RAs (n = 8,309). (**b**) Odds ratios were calculated comparing adverse event frequencies of PPI and H2RA patients. Abbreviations: CKD-Chronic Kidney Disease, AKI-Acute Kidney Injury, ESRD-End Stage Renal Disease, and NOS-Not otherwise specified. Ranges represent 95% confidence intervals (95% CI) (see Methods). X-axis is presented in log scale.

Patients who used PPIs with no other reported concurrent medications had a significant increase in the frequency of the following renal adverse event reports compared to the H2RAs: chronic kidney disease (CKD) (OR *28*.*4*, 95% CI [12.7, 63.5]), acute kidney injury (AKI) (4.2 [2.8, 6.3]), end-stage renal disease (ESRD) (35.5 [5.0, 250.0]), renal impairment of unspecified type (8.0 [5.0, 13.0]), and nephrolithiasis (2.8 [1.3, 6]) (Fig. 1b). The composite renal ADR frequency was 5.6% of the total PPI “monotherapy” ADRs reports and 0.7% for H2RA “monotherapy” reports (8.6 [6.6, 11]) (Fig. 1a).

**Figure 2:**
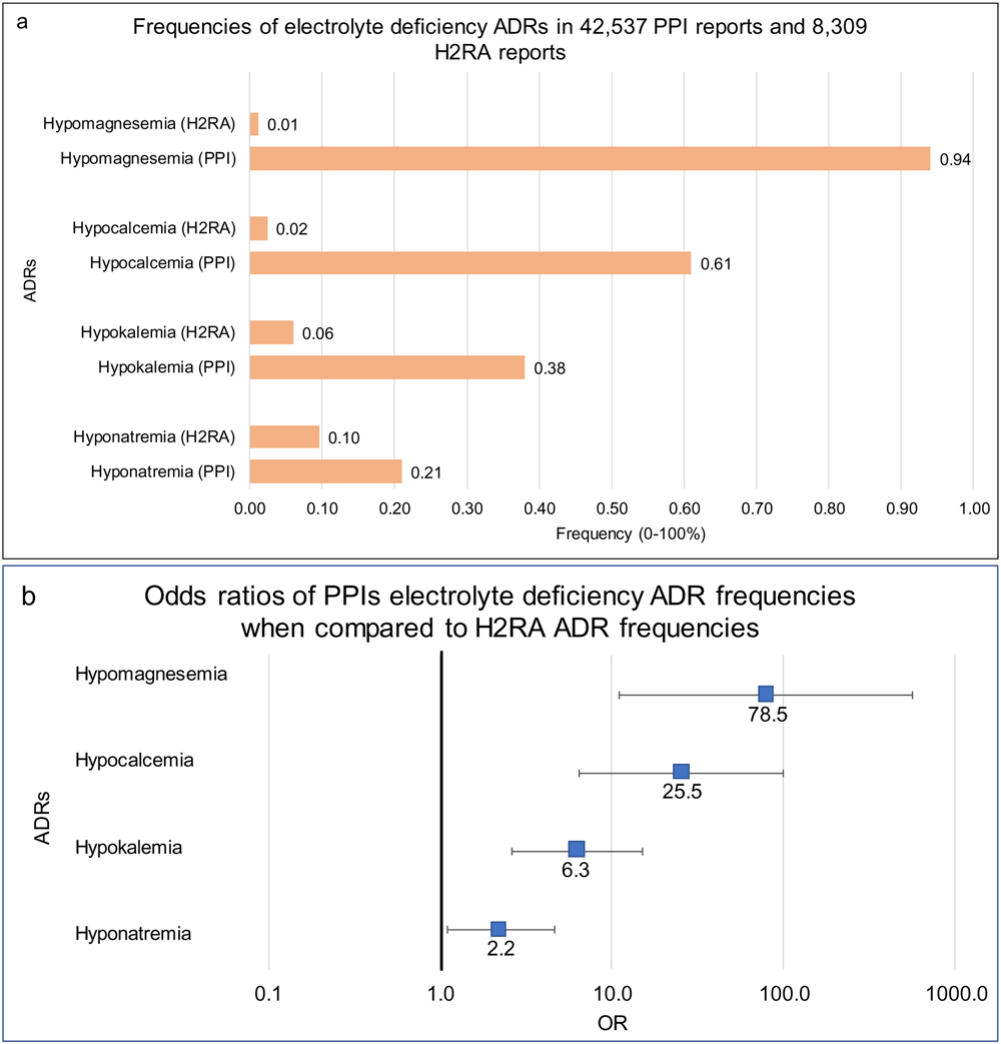
Frequencies and ORs of electrolyte related ADRs. (**a**) Frequencies of electrolyte related events for patients on PPIs (n = 42,537) and H2RAs (n = 8,309). (**b**) Odds ratios were calculated from adverse event frequencies. Ranges represent 95% confidence intervals (see Methods). X-axis is presented in log scale.

Interestingly, relative frequencies for electrolyte abnormalities followed the same trend (Fig. 2a) and were also increased for PPI users: hypomagnesemia (OR 78.5 [11, 560]), hypocalcemia (25.5 [6.4, 100]), hypokalemia (6.3 [2.6, 15]), and hyponatremia (2.2 [1.1, 4.6]) (Fig. 2b).

### Renal impairment and Individual PPIs

**Figure 3:**
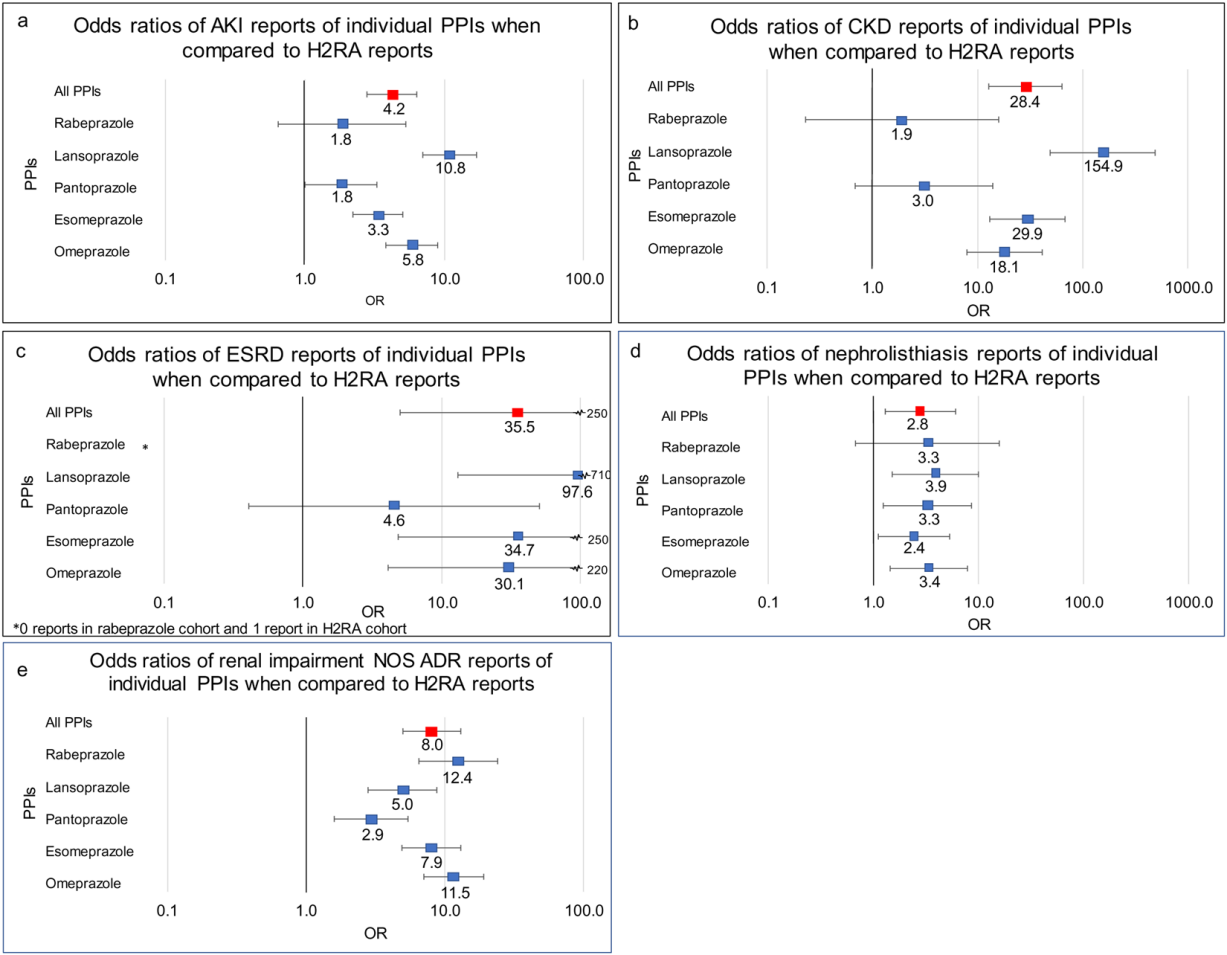
Renal ADR ORs of individual PPIs used as “monotherapy” (Rabeprazole n = 724, Lansoprazole n = 3,360, Pantoprazole n = 3,651, Esomeprazole n = 27,053, Omeprazole n = 7,749) when compared to H2RAs (n = 8,309). Odds ratios were calculated comparing (**a**) AKI (**b**) CKD, (**c**) ESRD (**d**) Nephrolithiasis and (**e**) renal impairment adverse event report frequencies of individual PPIs to all H2RAs (x-axis presented in log scale). NOS = not otherwise specified.

Analysis of renal and electrolyte adverse effects for each individual PPI produced the following results shown in Fig. 3.

### Acute Kidney Injury

Patients who received the following PPIs as “monotherapy” had a significant increase in the frequency of *AKI* reports: omeprazole (OR 5.8 [3.8, 8.9]), esomeprazole (3.3 [2.2, 5]), pantoprazole (1.8 [1.01, 3.3]), and lansoprazole (10.8 [7.0, 17]). Patients who received rabeprazole as “monotherapy” had an increase in *AKI* frequency, but it was not significant (1.8 [0.6, 5.3]) (Fig. 3a).

### Chronic Kidney Disease

Patients who received the following PPIs as “monotherapy” had a significant increase in the frequency of *CKD* reports: omeprazole (OR 18.1 [7.9, 41]), esomeprazole (29.9 [13, 67]), and lansoprazole (154.9 [49, 490]). Patients who received rabeprazole and pantoprazole as “monotherapy” had an increase in *CKD* frequency, but the significance criteria were not met (1.9 [0.2,16]) and (3.0 [0.7, 14]) respectively (Fig. 3b).

### End Stage Renal Disease

*ESRD* is of particular concern due to the limited prognosis in the absence of receiving dialysis or a kidney transplant. Very large OR values were determined for three widely used PPIs: omeprazole (OR 30.1 [4.1, 220]), esomeprazole (34.7 [4.8, 250]), and lansoprazole (97.6 [13, 710]) demonstrating a significant association with *ESRD*. The frequency of *ESRD* with pantoprazole “monotherapy” did not reach statistical significance (4.6 [0.4, 50]). Patients who received rabeprazole did not have any *ESRD* reports (Fig. 3c).

### Nephrolithiasis

Within the PPI cohort, patients who received the following PPIs as “monotherapy” had a significant increase in the frequency of *nephrolithiasis* reports: omeprazole (OR 3.4 [1.4, 7.9]), esomeprazole (2.4 [1.1, 5.3]), pantoprazole (3.3 [1.2, 8.6]), and lansoprazole (3.9 [1.5, 10.1]). Patients who received rabeprazole as “monotherapy” according to FAERS reports had an increase in *nephrolithiasis* frequency but did not meet the significance criteria (3.3 [0.7, 15.8]) (Fig. 3d).

### Renal Impairment

A large portion of renal impairment reports did not specify acuity of the injury, marked as *renal impairment NOS* (not otherwise specified). It was important to see if the observed renal side effects of PPIs persisted in this category of reports. In agreement with the preceding results, the OR values were significantly increased: omeprazole (OR 11.5 [7.1, 19]), esomeprazole (7.9 [4.9, 13]), pantoprazole (2.9 [1.6, 5.4]), lansoprazole (5.0 [2.8, 8.8]) and rabeprazole (12.4 [6.5, 24]) (Fig. 3e).

### Electrolyte Disturbances: magnesium, calcium, potassium, sodium

**Figure 4:**
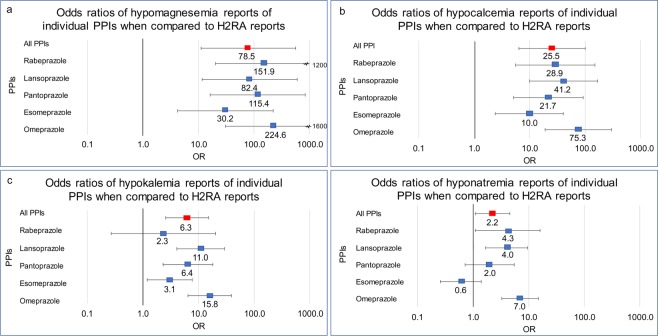
Electrolyte ADR odds ratios of individual PPIs used as “monotherapy” (Rabeprazole n = 724, Lansoprazole n = 3,360, Pantoprazole n = 3,651, Esomeprazole n = 27,053, Omeprazole n = 7,749) when compared to all H2RAs (n = 8,309). Odds ratios were calculated comparing (**a**) hypomagnesemia, (**b**) hypocalcemia, (**c**) hypokalemia, and (**d**) hyponatremia adverse event report frequencies of individual PPIs to all H2RAs.

All five PPIs were associated with a dramatic increase in *hypomagnesemia* reports (Fig. 4a, Table 1). Additionally, all the studied PPIs were associated with a significant increase in *hypocalcemia* reports (Fig. 4b, Table 1). Patients who received the following PPIs had an increase in the frequency of *hypokalemia* reports: omeprazole, esomeprazole, pantoprazole, and lansoprazole. Patients who received rabeprazole had an increase in *hypokalemia* frequency, but it was not statistically significant (Fig. 4c, Table 1).

**Table 1:** Electrolyte ADR odds ratios and 95% CIs of individual PPIs used as “monotherapy” (Rabeprazole n = 724, Lansoprazole n = 3,360, Pantoprazole n = 3,651, Esomeprazole n = 27,053, Omeprazole n = 7,749) when compared to all H2RAs (n = 8,309).

ADR	PPIs	OR	95% CI lower	95% CI upper
Hypomagnesemia	Omeprazole	224.6	31.0	1600.0
	Esomeprazole	30.2	4.2	220.0
	Pantoprazole	115.4	16.0	840.0
	Lansoprazole	82.4	11.0	600.0
	Rabeprazole	151.9	20.0	1200.0
Hypocalcemia	Omeprazole	75.3	19.0	300.0
	Esomeprazole	10.0	2.4	41.0
	Pantoprazole	21.7	5.1	93.0
	Lansoprazole	41.2	9.9	170.0
	Rabeprazole	28.9	5.6	150.0
Hypokalemia	Omeprazole	15.8	6.3	39.0
	Esomeprazole	3.1	1.2	7.7
	Pantoprazole	6.4	2.3	18.0
	Lansoprazole	11.0	4.1	29.0
	Rabeprazole	2.3	0.3	20.0
Hyponatremia	Omeprazole	7.0	3.3	15.0
	Esomeprazole	0.6	0.3	1.4
	Pantoprazole	2.0	0.7	5.5
	Lansoprazole	4.0	1.7	9.7
	Rabeprazole	4.3	1.1	16.0

Odds ratios were calculated comparing hypomagnesemia, hypocalcemia, hypokalemia, and hyponatremia adverse event report frequencies of individual PPIs to all H2RAs.

*Hyponatremia* was the least pronounced, yet still significant, electrolyte disturbance associated with omeprazole, lansoprazole, and rabeprazole. In contrast, reports of hyponatremia with esomeprazole or pantoprazole did not reach statistical significance. (Fig. 4d, Table 1).

## Methods

### FDA Adverse Event Reporting System

The FDA Adverse Event Reporting System (FAERS) supports FDA’s post marketing surveillance on drugs and biologic therapeutics submitted to FDA through MedWatch, the FDA Safety Information and Adverse Event Reporting Program. Reporting is voluntary and is done by doctors, pharmacists, legal representatives, other healthcare providers and patients. The manufacturer is the only contributor that is legally required to forward the information to the FDA.

Over 10.3 million FAERS/AERS reports, from January 2004 to March 2018, were used for the analysis. Data sets are available online at: https://www.fda.gov/Drugs/GuidanceComplianceRegulatoryInformation/Surveillance/AdverseDrugEffects/ucm082193.htm.

### Normalizing and combining the FAERS/AERS reports

Throughout the data set, the quarterly online reports were not homologous from year to year. Each quarterly set was downloaded in dollar-separated text format (.txt) and modified, standardized, and extended. Missing columns in data sets were added with no values and the column names were homogenized. The final data set version contained over 10.3 million reports. Most of the reports were submitted from the United States, and many were submitted from all around the world with their respective country specific demographic formats. Online drug databases were used to generate a dictionary with all international brand/generic drug names and to translate them into generic names.

### Study outcomes

A fraction of the observed 20,317 outcomes in FAERS and AERS were grouped into the following generalized outcomes for *a priori* hypothesis: chronic kidney disease (CKD: defined in FAERS and AERS data sets as chronic kidney disease, and chronic renal failure), acute kidney injury (AKI: acute kidney injury, acute prerenal failure, renal failure acute), and end-stage renal disease (ESRD: end stage renal disease, end stage kidney disease), nephrolithiasis, renal impairment NOS (renal failure, renal impairment, renal disorder, renal injury), hypomagnesemia (hypomagnesemia, decreased blood magnesium), hypocalcemia (hypocalcemia, decreased blood calcium), hypokalemia (hypokalemia, decreased blood potassium) and hyponatremia (hyponatremia, decreased blood sodium).

### Choice of cohorts

A total of 10,324,033 FAERS and AERS reports were collected. Reports where omeprazole, esomeprazole, rabeprazole, lansoprazole, pantoprazole, and dexlansoprazole were used, excluding reports with concurrent use of any H2RAs, were selected into the PPI cohort (n = 732,696). Reports where ranitidine, famotidine, cimetidine, and nizatidine were used, excluding reports with concurrent use of any PPI, were selected into the H2RA cohort (n = 162,189). Further, reports where PPIs and H2RAs were used as *monotherapy* were selected into the respective cohorts. The term “monotherapy” pertains to records where PPIs and H2RAs were the only reported medications. PPIs-only cohort comprised of 42,537 reports and H2RA-only cohort comprised of 8,309 reports.

**Figure 5:**
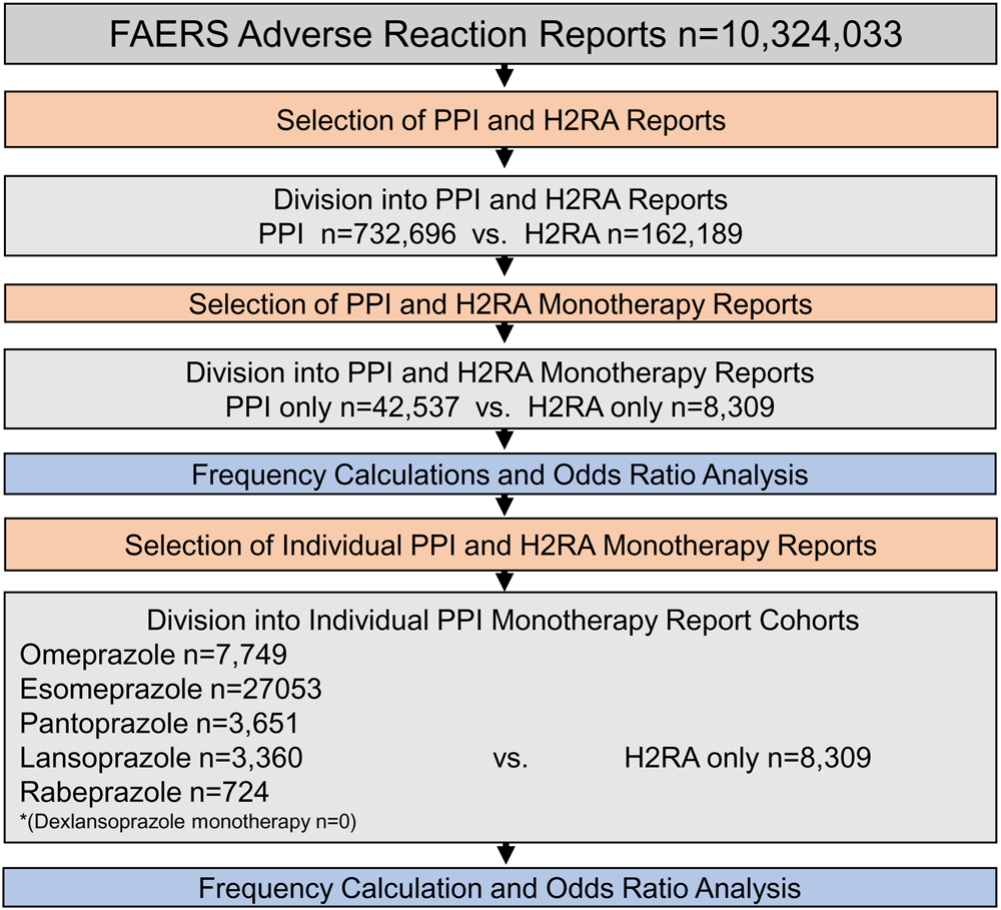
Legend: Inclusion exclusion and analysis cohort selection for adverse event rate comparison between PPIs and H2Ras as a class, as part of therapeutics, “monotherapy”, and between individual PPIs and all H2RAs.

Odds ratio analysis was performed by comparing the ADR frequencies of PPIs in relation to H2RA “monotherapy” frequencies. The PPI “monotherapy” cohort was further split into individual PPI cohorts which included omeprazole (n = 7,749) esomeprazole (n = 27,053), pantoprazole (n = 3,651), lansoprazole (n = 3,360), and rabeprazole (n = 724). There were no reports where dexlansoprazole was used as “monotherapy”. Reports with two or more PPIs used were excluded (Fig. 5). Demographic analysis was performed (Tables 2 and 3). Overall distributions strongly overlap and validate the cohort choice. Each individual PPI cohort frequency of renal and electrolyte ADRs was calculated and compared to the H2RA cohort to screen for potential ADR variability within individual PPIs in the cohort (Figs 3 and 4).

**Table 2:** Frequency of PPI and H2RA “monotherapy” reports in FAERS by country of origin.

Country	No. of PPI reports	Frequency %	No. of H2RA reports	Frequency %	% Difference	>1% Difference
United States	37,139	88.26	6,928	87.27	0.99	
Great Britain	1122	2.67	177	2.23	0.44	
Japan	472	1.12	382	4.81	3.69	*
Germany	357	0.85	24	0.30	0.55	
France	330	0.78	29	0.37	0.42	
Canada	309	0.73	22	0.28	0.46	
Italy	312	0.74	39	0.49	0.25	
Brazil	282	0.67	3	0.04	0.63	
Turkey	173	0.41	13	0.16	0.25	
Australia	151	0.36	7	0.09	0.27	
China	149	0.35	9	0.11	0.24	
Denmark	143	0.34	4	0.05	0.29	
Spain	138	0.33	13	0.16	0.16	
Nederlands	103	0.24	34	0.43	0.18	
Sweden	52	0.12	6	0.08	0.05	
Singapore	48	0.11	9	0.11	0.00	
Belgium	41	0.10	9	0.11	0.02	
New Zealand	42	0.10	3	0.04	0.06	
Chile	28	0.07	3	0.04	0.03	
India	15	0.04	15	0.19	0.15	
Costa Rica	30	0.07	0	0.00	0.07	
Unknown	198	0.47	45	0.57	0.10

**Table 3:** Patient demographics in PPI and H2RA “monotherapy” cohorts.

Sex	PPI reports (n = 42,537)	Frequency (%)	H2RA reports (n = 8,309)	Frequency (%)	P-value	% Difference
Female	25,116	59.69	4,579	57.68	<0.001	2.01
Male	12,000	28.52	2,710	34.14	<0.001	5.62
Unreported	4,963	11.79	650	8.19	<0.001	3.61
						Age difference
Mean age, years (SD)	58.3 (15.9)		55.6 (20.1)		<0.001	2.7
Median age	58.6		59.7		<0.001	1.1
Unreported (%)	45.4		55.1			

### Statistical analysis

#### Descriptive Statistics

Frequencies for each studied side effect (Figs 1a and 2a) was calculated by the equation:

1 $$Frequency=(Number\,of\,Records\,with\,ADR)/(Number\,of\,Patient\,Records)\ast 100$$

#### Comparative Statistics

ADR report rates were compared via the Odds Ratio (OR) analysis for Figs 1b, 2b, 3 and 4 using the following equations:

2 $$OR=(a/b)/(c/d)$$

where

a: Number of cases in exposed group with an adverse event.

b: Number of cases in exposed group with no adverse event.

c: Number of cases in control group with the adverse event.

d: Number of cases in control group with no adverse event.

3 $${\rm{LnOR}}=\,\mathrm{Ln}({\rm{OR}})$$

4 $$Standard\,Error\,of\,Log\,Odds\,Ratio;\,SE(LnOR)=\sqrt{(1/a+1/b+1/c+1/d)}$$

95% Confidence Interval;

5 $$95 \% \,CI=exp\,(LnOR-1.96\times S{E}_{LnOR})\,to\,exp\,(LnOR+1.96\times S{E}_{LnOR})$$

## Discussion

In this study, we quantified and confirmed the association between PPI exposure and the increased risk of AKI, CKD, ESRD, and electrolyte abnormalities by utilizing updated adverse event reports in the FAERS/AERS database. Most interestingly, the extended set of reports revealed an association between PPI exposure and unexpected significant risk for nephrolithiasis and renal impairment (Fig. 1). For the first time there were sufficient records for analysis of the effects of individual PPIs and observed varying degrees of electrolyte and renal abnormalities (Figs 3 and 4).

Our analysis of renal adverse effects was in general agreement with previous studies that have documented an increased risk of incident AKI, incident CKD, CKD progression and ESRD in large observational cohorts. Klesper and colleagues performed a nested case-control study, including 184,480 patients, and found an increased risk of AKI with PPI prescription (OR 1.72, 95% confidence interval [CI] [1.27, 2.32], p < 0.001)^22^. In a population-based cohort study of 290,593 patients over the age of 65, Antoniou and colleagues confirmed the association of PPI use with AKI (hazard ratio [HR] 2.52, 95% CI [2.27, 2.79])^29^. In the Atherosclerosis Risk in Communities (ARIC) study Lazarus *et al*. performed a population based prospective cohort study with 10,482 patients and found an increased risk of incident AKI and CKD when comparing PPI users to H2RA users (HR, 1.58; 95% CI [1.05, 2.04]) and (HR, 1.39; 95% CI [1.01, 1.91]), respectively)^21^. These population-based studies utilized ICD coding data to define the outcome of incident AKI and CKD. Xie and colleagues evaluated a prospective cohort including 193,591 patients from the Veteran’s Affairs database and documented not only an increased risk of incident CKD (HR, 1.28; 95% CI [1.23, 1.34]) but also an increased risk for ESRD when comparing PPI users to H2RA users (HR, 1.96; 95% CI [1.21, 3.18])^23^ and in a later study demonstrated that CKD progression and ESRD can occur without intervening AKI^28^. The difference in values between different studies may be due to multiple factors including definitions of renal injury and the diagnostic criteria as well as the time dependent analyses using hazard ratios. The FAERS and AERS data derived frequencies are additionally influenced by the severity related threshold of the report submission. In summary we documented an OR of 4.2 for AKI with the lower 95% CI boundary of 2.9, OR values as large as 28.4, with the 95% CI between 12.7 and 63.5 for CKD and 35.5 with 95% CI between 5.0 and 250.0 for ESRD. Our results indicate significant increase in nephrolithiasis reports with (OR 2.8 (95% CI [1.3, 6.0]). Nephrolithiasis finding is of particular interest since it has been associated with AKI, CKD, and ESRD progression but it only accounts for a small subset of renal injury cases^30–32^.

Hypomagnesemia was reported in the initial clinical trials and on the FDA package insert for every PPI as a rare side effect^11^. Accordingly, the frequency of hypomagnesemia reports for PPI patients is low, but the relative frequency was dramatically higher, almost eighty-fold, than for the H2RA control group. Secondly, detection bias may underestimate this adverse effect, since magnesium concentrations are not routinely measured compared to sodium, potassium and calcium. All five studied PPIs had comparable and increased ORs, with omeprazole showing the largest magnitude. Omeprazole, being the first marketed and the first over-the-counter PPI, has been used for the longest time, therefore patients were likely to have longer drug exposure. It may be prudent to monitor magnesium levels in patients with ongoing PPI therapy and other risk factors for hypomagnesemia.

Previous studies examining the effect of PPIs’ on calcium levels were not consistent. Multiple small-scale studies have shown that PPIs decrease gastrointestinal calcium absorption^33–35^ and this deleterious effect is attenuated by administering acidic liquids^36^. However, other studies have noted that the change in gastric pH does not correlate with calcium levels^37,38^. In our analysis of 42,537 PPI and 8,309 H2RA cases, we found a clear increase in hypocalcemia in patients taking PPIs compared to patients receiving H2RAs. Although the mechanism for hypocalcemia is not clearly defined, we can conclude that all PPIs are significantly associated with hypocalcemia.

We evaluated alterations in serum potassium concentrations. The previous evidence for hypokalemia with PPI use was limited, mainly consisting of case reports^39–41^. We found that PPI utilization resulted in moderately increased hypokalemia, when compared to the H2RA cohort. As noted earlier, PPI utilization was correlated with CKD, known to cause fluctuations in electrolytes. While CKD can be associated with both hypokalemia (e.g. in the case of tubular dysfunction) and hyperkalemia^42^, we only observed a significant increase in hypokalemia. However, a small number of cases of hyperkalemia were reported out of 42,537 reports in FAERS/AERS. In conclusion, hypokalemia was more common than hyperkalemia in our analysis of patients receiving PPIs. Each PPI was shown to have increased odds of hypokalemia, except for rabeprazole which was not significant (OR 2.3 CI [0.27, 20]).

Hyponatremia has been reported as a rare post marketing adverse reaction in FDA package inserts for pantoprazole, omeprazole, and esomeprazole^11,43,44^. In a retrospective study of 302 individuals receiving PPIs for longer than a year, Buon and colleagues found moderate hyponatremia in 18.7–46.3% of elderly patients^45^. It should be noted that although dysnatremia is associated with CKD^46^, we observed only a minor yet significant increase in hyponatremia reports. Hypernatremia was not a significant adverse effect in PPI users. Our analysis showed hyponatremia effect to be most pronounced for omeprazole, followed by lansoprazole and rabeprazole.

The observed increased risks of renal and electrolyte adverse effects of PPIs warrant more careful consideration in clinical practice. The risk-benefit ratio should be considered for the individual patient with respect to the adverse effects. When clinically indicated, PPIs should be used for the shortest duration necessary and chronic use is not recommended except for treatment of pathological hypersecretory conditions including Zollinger-Ellison syndrome and maintenance healing of erosive esophagitis^11,43,44,47–54^. It should be noted that the above-mentioned indications are FDA recommendations. Off-label and over-the-counter use of PPIs for the treatment of gastroesophageal reflux disorder (GERD) should be limited to four weeks^11,43,44,52–54^ but is often continued beyond the recommended limit. Continued use can result in rebound acid hypersecretion and hypergastrinemia after 4–8 weeks of therapy^55,56^ leading to chronic use.

## Conclusion

In our study we observed various levels of increased risk of renal and electrolyte ADRs in FAERS reports of individual PPI drugs with respect to H2RA reports. Regardless of which PPI is initiated, it may be beneficial to follow dosing and duration recommendations established by the FDA^11,43,44,52–54^, American College of Gastroenterology^47,48^ and World Gastroenterology Organisation Global Guidelines^49^. It may be beneficial to monitor renal function and electrolytes including potassium, calcium, magnesium, and sodium. Although H2RAs have not been shown to be as effective as PPIs, they might be considered as alternatives for patients who are at high risk for developing renal and electrolytes imbalances.

## Study Limitations

Since the FDA FAERS/AERS reporting is voluntary, only a subset of actual cases is represented, and ADR frequencies do not represent the population incidences. A recent study found that FAERS/AERS reporting can be biased by legal and scientific variables and newsworthiness^50^. Another study has shown that FAERS/AERS reporting was significantly underreported for statins^51^. Some limitation stems from the absence of comprehensive medical records. Although the indication section in the data set was used to exclude potential comorbidities, some concurrent medications and comorbidities may be missing from the records due to underreporting. This may have introduced noise into the cohort compositions, ADR frequencies and odds ratios. The mechanism of the adverse reaction cannot be derived from the FAERS/AERS records. The odds ratios represent frequency ratios of reported adverse effects between FAERS/AERS PPI and H2RA cohorts and are not based on population incidences. As with any association study, causality cannot be inferred from association. These cases were not clinically adjudicated for causality by experts.

## Supplementary information

Supplementary Information, Appendix A
