# Supplementary material for: Analysis of postmarketing safety data for proton-pump inhibitors reveals increased propensity for renal injury, electrolyte abnormalities, and nephrolithiasis
Source: Sci Rep. 2019 Feb 19;9:2282. doi: 10.1038/s41598-019-39335-7 (PMC6381091; doi:10.1038/s41598-019-39335-7)
Supplement: Supplementary file 1 — Supplementary Information, Appendix A [file 41598_2019_39335_MOESM1_ESM.pdf]

Analysis of postmarketing safety data for proton-pump inhibitors reveals increased propensity for renal injury, electrolyte abnormalities, and nephrolithiasis.

Tigran Makunts[1], Isaac V. Cohen[1], Linda Awdishu[1], Ruben Abagyan[1,*]

[1]Skaggs School of Pharmacy and Pharmaceutical Sciences, University of California, San Diego, La Jolla CA, 92093-0747.

* Corresponding author, ruben@ucsd.edu

Supplementary Information, Appendix A

PPI and H2RA Indications as Reported to FAERS/AERS

*Notice partial duplication of terms due to difference in U.K. and U.S. spelling.

Gastrointestinal disorder, gastrointestinal stromal tumour, gastrointestinal haemorrhage, gastrooesophageal reflux prophylaxis, gastrointestinal disorder therapy, gastrointestinal motility disorder, gastrointestinal ulcer, gastrointestinal pain, gastroduodenal ulcer, gastroenteritis, upper gastrointestinal, haemorrhage, gastrooesophagealcancer, gastrointestinalcarcinoma, gastroduodenitis, gastrointestinal inflammation, functional gastrointestinal disorder, gastrointestinal ulcer haemorrhage, gastroesophageal reflux disease, gastrooesophageal sphincter insufficiency, gastrointestinal tract irritation, gastrointestinal mucosal disorder, gastrointestinal infection, gastrointestinal hypomotility, gastroesophageal reflux, gastrointestinal erosion, gastrooesophagitis, gastrointestinal neoplasm, gastrointestinal angiodysplasia, gastrooesophageal reflux, gastrointestinal ulcer management, gastrointestinal stoma complication, gastroesophageal reflux prophylaxis, lower gastrointestinal haemorrhage, gastrointestinal stromal tumor,gastrointestinal oedema, gastrointestinal obstruction, gastrointestinal necrosis, gastrointestinal mucosa hyperaemia, gastrointestinal injury, gastrointestinal cancer metastatic, gastroenteritis paracolon bacillus, gastroenteritis helicobacter, distress gastrointestinal, gastrooesophageal reflux disease, barrett's oesophagus, oesophagitis, reflux oesophagitis, oesophageal disorder, erosive oesophagitis, oesophageal ulcer, gastrooesophageal reflux prophylaxis, oesophageal carcinoma, oesophageal spasm, eosinophilic oesophagitis , oesophageal pain, varices oesophageal, oesophageal stenosis, oesophageal candidiasis, oesophageal adenocarcinoma, gastrooesophageal cancer, oesophageal achalasia, oesophagitis ulcerative, oesophageal discomfort, oesophageal cancer metastatic, oesophageal irritation, gastroesophageal reflux disease, oesophageal haemorrhage, gastrooesophageal sphincter insufficiency, dyskinesia oesophageal, gastroesophageal reflux, burn oesophageal, acid reflux (oesophageal), radiation oesophagitis, oesophageal rupture, oesophageal motility disorder, oesophageal dysplasia, oesophageal adenocarcinoma metastatic, oesophagitis haemorrhagic, oesophageal varices haemorrhage, gastrooesophagitis, gastrooesophageal variceal haemorrhage prophylaxis, tracheo-oesophageal fistula, oesophagram, oesophagogastric fundoplasty, oesophageal ulcer haemorrhage, oesophageal squamous cell carcinoma, oesophageal

perforation, oesophageal oedema, oesophageal obstruction, oesophageal injury, oesophageal dilatation, oesophageal atresia, gastrooesophageal reflux, gastroesophagealreflux prophylaxis, diverticulitis oesophageal, oesophagogastroduodenoscopy, oesophageal mucosal tear, oesophageal infection, oesophageal adenocarcinoma recurrent, fungal oesophagitis, gerd, antacid therapy, acid reflux, acidosis, acid peptic disease, acid dyspepsia, gastrooesophageal reflux disease, reflux oesophagitis, reflux gastritis, gastrooesophageal reflux prophylaxi, reflux laryngitis, duodenogastric reflux, gastroesophageal reflux disease, gastroesophageal reflux, gastrooesophageal reflux, gastroesophageal reflux prophylaxis, gastric ulcer, colitis ulcerative, prophylaxis against gastrointestinal ulcer, ulcer, peptic ulcer, duodenal ulcer, oesophageal ulcer, ulcer haemorrhage, gastric ulcer haemorrhage, gastrointestinal ulcer, gastroduodenal ulcer, stress ulcer, gastric ulcer helicobacter, oesophagitis ulcerative, duodenal ulcer haemorrhage, gastrointestinal ulcer haemorrhage, scleroderma associated digital ulcer, perforated ulcer, peptic ulcer haemorrhage, gastric ulcer perforation, stomach ulcer, anastomotic ulcer, peptic ulcer helicobacter, oesophageal ulcer haemorrhage, gastrointestinal ulcer management, duodenal ulcer perforation, decubitus ulcer, aphthous ulcer, non-ulcer dyspepsia, neuropathic ulcer, necrotising ulcerative gingivostomatitis, large intestinal ulcer, gastric ulcer prophylaxis, duodenal ulcer, obstructive, helicobacter infection, helicobacter gastritis, gastric ulcer helicobacter, helicobacter test positive, helicobacter pylori infection, peptic ulcer helicobacter, helicobacter pylori identification test positive, helicobacter pylori gastritis, gastroenteritis helicobacter, helicobacter pylori identification test positive, bacterial infection due to helicobacter pylori.
